# **Inclusion body myositis, viral infections, and TDP-43**: **a narrative review**

**DOI:** 10.1007/s10238-024-01353-9

**Published:** 2024-05-02

**Authors:** Vitalie Văcăraş, Romana Vulturar, Adina Chiş, Laura Damian

**Affiliations:** 1 Department of Neurosciences, “Iuliu Haţieganu” University of Medicine and Pharmacy, Cluj-Napoca, 43, Victor Babeş St, 400012 Cluj-Napoca, Romania; 2 Neurology Department of Cluj, County Emergency Hospital, 3-5, Clinicilor St, 400347 Cluj-Napoca, Romania; 3 Department of Molecular Sciences, “Iuliu Haţieganu” University of Medicine and Pharmacy Cluj-Napoca, 6, Pasteur St, 400349 Cluj-Napoca, Romania; 4 Cognitive Neuroscience Laboratory, University Babeş-Bolyai, 30, Fântânele St, 400294 Cluj-Napoca, Romania; 5 Association for Innovation in Rare Inflammatory, Metabolic, Genetic Diseases INNOROG, 30E, Făgetului St, 400497 Cluj-Napoca, Romania; 6 Department of Rheumatology, Centre for Rare Autoimmune and Autoinflammatory Diseases, Emergency, Clinical County Hospital Cluj, 2-4, Clinicilor St, 400006 Cluj-Napoca, Romania; 7 CMI Reumatologie Dr. Damian, 6-8, Petru Maior St, 400002 Cluj-Napoca, Romania

**Keywords:** TDP-43, Inclusion body myositis, Myositis triggers, Interferon gamma, Long COVID

## Abstract

The ubiquitous RNA-processing molecule TDP-43 is involved in neuromuscular diseases such as inclusion body myositis, a late-onset acquired inflammatory myopathy. TDP-43 solubility and function are disrupted in certain viral infections. Certain viruses, high viremia, co-infections, reactivation of latent viruses, and post-acute expansion of cytotoxic T cells may all contribute to inclusion body myositis, mainly in an age-shaped immune landscape. The virally induced senescent, interferon gamma-producing cytotoxic CD8+ T cells with increased inflammatory, and cytotoxic features are involved in the occurrence of inclusion body myositis in most such cases, in a genetically predisposed host. We discuss the putative mechanisms linking inclusion body myositis, TDP-43, and viral infections untangling the links between viruses, interferon, and neuromuscular degeneration could shed a light on the pathogenesis of the inclusion body myositis and other TDP-43-related neuromuscular diseases, with possible therapeutic implications.

## Background

Inclusion body myositis (IBM) is an inflammatory myopathy occurring after middle age, with autoimmune and degenerative mechanisms [1, 2]. Other idiopathic inflammatory myopathies (IIMs) are dermatomyositis (DM), polymyositis (PM), overlap syndromes including anti-synthetase syndrome and necrotizing pauci-immune myositis [3]. The distinction between IBM, PM, and PM with mitochondrial pathology is not neat, raising the question whether IBM is a variant of PM occurring in the older age, related to immunosenescence [4]. IBM pathogenesis centrally involves cytotoxic, senescent CD8+ T cells, defects of autophagy and ubiquitin–proteasome system (UPS) resulting in proteostasis impairment and abnormal sarcoplasmic protein aggregation, along with endoplasmic reticulum and mitochondrial alterations, and antibodies to the cytosolic 5′-nucleotidase 1A (anti-cN1A) [1, 5]. The driving mechanisms of this pathology, however, are still evasive.

IBM belongs to a group of neurological disorders, the TDP-43 proteinopathies, which pathogenically involve TDP-43 [TAR-DNA-binding protein 43 (transactive response DNA-binding protein of 43 kDa)] [6]. TDP-43, encoded by the *TARDBP* gene, an RNA- and DNA-binding nuclear regulatory protein, member of the heterogeneous nuclear ribonucleoprotein (hnRNP) family [7, 8]. In skeletal muscles, TDP-43 is involved in transcription regulation, RNA splicing, mRNA stability, RNA transport, and quality control and undergoes post-translational modifications with functional consequences [9]. TDP-43 functions in muscles are complex, including myoregeneration (Table 1). In neurodegeneration, the mechanisms of TDP-43 involvement include cytotoxic aggregations, nuclear loss, alteration of cellular functions, and others [6, 10].

**Table 1:** Roles of TDP-43 in muscles

Function	Roles of TDP-43	References
mRNA metabolism	TDP-43 is involved in transcription regulation, nucleocytoplasmic shuttling, mRNA splicing, translation, transcription, transport, stabilization, miRNA, and lncRNA processing, and RNA quality control TDP-43 binds ssRNA and DNA and acts as a transcription repressor, or scaffold for nuclear bodies	[1–3, 9, 11–14]
Myogenesis	TDP-43 is involved in muscle development and differentiation, neuromuscular junction formation, and muscle regeneration after injury TDP-43 transiently forms during myogenesis amyloid-like myogranules, along with RNA- and RNA-binding proteins TDP-43 is required for the expression of myogenesis regulators and myogenic microRNAs such as miR-1 and miR-206 [9, 13] TDP-43 activates Wnt/β-catenin signaling, involved in muscle regeneration and fibrosis	[5, 6, 9, 13, 15–18]
Association with mitochondria	In myogranules, TDP-43 co-localizes and interacts with the mitochondrial inner membrane protein CHCHD10 . In IBM, TDP-43 aggregates accumulate with mitochondria in myofibers, resulting in mitochondrial and muscle fibers toxicity. TDP-43 targets the mitochondria complex I	[6, 19, 20]

*IFN* interferon, *RBP* ribonucleoproteins, *lncRNA* long non-coding RNAs, *ssRNA* single-strand RNA

IBM muscle biopsies reveal cytoplasmic aggregation of TDP-43 and TDP-43 nuclear loss [10]. Even an 1% amount of myofibers staining for TDP-43 in a muscle biopsy was highly sensitive and specific for IBM [11].

TDP-43 may have an emerging intriguing role in viral infections [12]. TDP-43 is involved in controlling IFN responses triggered by endogenous RNA, but the TDP-43 role as an RNA-binding protein in viral infections is rarely investigated [13, 14]. Loss of TDP-43 results in dsRNA intracellular accumulation and interferon (IFN) triggering [13]. The TDP-43 ortholog of *Caenorhabditis elegans* called TDP1 limits dsRNA accumulation [21]. Also, knockdown of *TARDBP* increases viral replication in macrophages [14] and TDP-43 knockdown amplifies enterovirus infections, suggesting an antiviral effect of TDP-43 [22]. Moreover, TDP-43 binding is protective against HIV-1 by sterically hindering a HIV-1 promoter [23]. Also, after TDP-43 knockdown in mouse brain, the type I IFN-inducible genes, including the mouse orthologs of the intracellular sensor molecules RIG-I and MDA-5 which detect viral RNA, are the most overexpressed [21, 24]. In *Coxsackie* B3 infection, the viral protease 2A alters TDP-43 distribution, solubility, and function [22]. Therefore, TDP-43 could have an important role in the viral-induced IFN response in TDP-43 proteinopathies, including IBM (Table 2).

**Table 2:** Immunomodulatory and antiviral roles of TDP-43

Function	Roles of TDP-43	References
Immunomodulatory	TDP-43 regulates the accumulation of RNA polymerase III transcripts and other endogenous immunostimulatory dsRNAs which trigger IFN TDP43 limits overexpression of IFN-I related genes including RIG-I and MDA-5 in animal models TDP-43 interacts with lncRNAs such as *Malat1*, which prevents TDP-43 cleavage and IFN generation TDP-43 aggregation may be induced by IFN-ƴ and by low amounts of cytoplasmic RNA TDP-43 expression activates GSK3, which delays and decreases IFN-1 production and enhances IFNγ and other pro-inflammatory cytokines production GSK is involved in TDP-43 phosphorylation and aggregation	[9, 11, 25–34]
Antiviral	TDP-43 binds YB-1, a host regulator of HCV replication	[35]
	TDP-43 suppresses HIV1 transcription by binding HIV-1 long terminal repeats HIV-1 could replicate in human immune cells independent of TDP-43 A specific deubiquitinase inhibitor, IU1, reversed HIV-1 latency by degrading TDP-43 Knocking down TDP-43 with siRNAs in cell cultures reactivates HIV-1 by reversing its latency TDP-43 binding may sterically hinder the HIV-1 LTR promoter involved in viral transcription and reactivation Silencing TDP-43 increases HIV-1 infectivity by reducing HDAC6	[12, 36, 37] [23]
	TDP-43 is protective against enteroviruses	[38]
	TDP-43 RRM binds the SARS-CoV2 S1 RBD	[39]

*dsRNA* double-strand RNA, *IFN* interferon, *RBP* ribonucleoproteins, *lncRNA* long non-coding RNAs, *Malat1* metastasis-associated lung adenocarcinoma transcript-1, *GSK3* glycogen synthase kinase 3, *RRM* RNA recognition motif 1, *SARS-CoV2 S1 RBD* SARS-CoV-2 spike S1 protein receptor binding domain, *RBPs* RNA-binding proteins, *YB-1*-box-binding protein-1

## Role of TDP-43

### TDP-43 in basal conditions and in infections

In basal conditions, TDP-43 is bound by the long-non-coding RNA (lncRNA) Malat1 (metastasis-associated lung adenocarcinoma transcript-1), in humans called MALAT1 [14]. Malat1 binding hinders the TDP-43 cleavage, mediated by activated caspase-3, from generating TDP-35 and IRF3 (IFN regulator factor 3) [14]. Generally, viral infections result in reduced expression of Malat1, promoting antiviral IFN production [14]. Moreover, TDP-35 amplifies the IFN–I production by degrading the negative regulator of IRF3 called Rbck1 (RanBP-type and C3HC4-type zinc finger-containing protein 1) [14]. However, Malat1 function may increase in certain viral infections, such as HIV, *Coxsackie* myocarditis or mild COVID-19 [14, 25, 40]. Malat1 has immunosuppressor and NF-kB- and NLRP3 regulatory effects [40, 41]. Also, MALAT1 is upregulated in IBM [42]. Increased Malat1 in IBM and TDP-43 aggregation may likely depend on viral characteristics and is in line with a slow inflammatory response.

TDP-43 can be activated after caspase-induced cleavage, the N-terminal cleavage product of TDP-43 forming protein aggregates, while the C-terminal cleavage product is degraded by proteasomes [22]. The proteasome inhibition contributes to the pathogenesis of IBM, as the major proteasomal enzymes have decreased activity [43]. Immunoproteasomes (iPS) found in immune tissues (constitutively expressed in hematopoietic cells or induced in response to IFN gamma or TNF alpha) have structural similitudes to proteasomes but have three different inducible catalytic subunits (PSMB8, -B9, and -B10), triggered by IFN-ƴ in viral infections, or by other pathogens, proteins, or particles [44–46]. Generation of cytotoxic CD8+ T cell responses upon a viral infection requires antigen processing through the proteasome, which selectively cleaves after certain ammino acids residues [46].

### TDP-43 in IBM biopsies

In IBM muscle biopsies, the overexpression of the immunoproteasome (iPS) subunits PSMB8 and-9, correlated with IFN-ƴ, IRF1 (interferon regulatory factor 1), and STAT1 (signal transducer and activator of transcription 1), is another argument for a viral trigger [44, 47]. The iPS upregulates the major histocompatibility complex MHC-1 and MHC-2 on myofibers, exposing them to immune attack [48–50]. Also, iPS are involved in muscle remodeling and prevention of protein aggregation [51]. Of note, mutations of the (immuno)proteasome subunits, as in the rare autoinflammatory diseases Nakajo–Nishimura or CANDLE syndrome, may result in an IBM-like myositis [52].

IBM patients have a high IFN score and IFN-ƴ signature, along with increased IFN type I expression in muscle which amplifies inflammation [53, 54]. The IFN-ƴ, central in IBM, is produced by the highly differentiated cytotoxic CD8+ T cells, reprogrammed with aging to fulfill innate-like functions [55, 56]. These CD8+ cells and effector memory T cells re-expressing CD45RA (TEMRA) found in IBM may be induced not only by senescence, but also by persistent viruses [1, 4, 55, 57]. IFN-ƴ induces ER stress and aggregation of TDP-43 and other proteins [1, 5, 54]. IFN-I may also be induced by anti-Ro52, present in some IBM patients [50, 58]. Ro52 or TRIM21 (tripartite motif proteins), is an IFN–inducible E3 ligase involved in IFN type I downregulation [59]. Other infection-related factors may intervene in IBM, such as activation of NLRP3 inflammasome, heat shock proteins (HSP), ribosomal proteins, or molecular mimicry with a *mycobacterial* protein guanylate-binding protein 2 (GBP2) with antiviral and anti-tuberculous functions [5, 60]. GBP2 is involved in the control of mRNA splicing [5] with possible relevance in TDP-43 dysfunction when mRNA splicing is altered.

Also, the glycogen synthase kinase 3 (GSK3), a serine/threonine kinase with 2 isoforms (α and β), is activated in IBM [33]. GSK3, involved in many cellular processes, is an immunomodulator in IBM [33]. GSK3 delays and decreases IFN-1 production, enhances IFNγ signaling, but also increases and delays pro-inflammatory cytokines production [33]. Moreover, GSK3β is one of the protein kinases involved in the TDP-43 phosphorylation [34]. TDP-43 expression activates GSK3, and GSK inhibition decreases TDP-43 aggregation [35].

Also, activation of autophagy is part of the innate immune response, and autophagy receptors may become viral targets [15]. Amongst these autophagy receptors, NBR1 (neighbor of BRCA1), a ubiquitin-binding scaffold protein, increases in viral infections [61], and NBR1 accumulates and is abnormal in IBM muscle [62].

In IBM, the dysregulation of a deubiquitinase called cylindromatosis (CYLD) reduces the autophagic clearance of protein aggregates [63]. CYLD is expressed with phosphorylated TDP-43 in the sIBM myofibers [63]. CYLD, required for antiviral host defense, is involved in the STING cleavage [64] and negatively regulates NF-kB [63].

IFN-ƴ and low RNA amounts in cytoplasm also stimulate aggregation of TDP-43 and other RBPs with “prion-like” low-complexity (LC) domains, favored by proteins misfolding in aging [1, 65].

### Potential links between IBM and viral infections

The IBM occurrence may reflect various pathogenic associations, including viral infections [4]. In general, chronic IIM may be triggered by viruses such as *Coxsackie B*, enterovirus, parvovirus, HTLV-1, or HIV [66]. Mechanisms of viral-induced myositis hypothetically include direct invasion of myocytes by the virus, molecular mimicry, exposure of cryptic epitopes after conformational alterations, myotoxic cytokines such as IFNs and autoimmune reactions [66–68]. Latent viral infection, viral-induced denaturation of self-structures or homologies with various viral proteins could result in a prolonged immune response [66]. For instance, enterovirus 71 (EV71) may upregulate TRIM21 (Ro52), which degrades SAMHD1, a host antiviral molecule [59]. Also, during a viral infection, many ribonucleoproteins including TDP-43, are hijacked [12]. Coxsackie virus B3 protease 3C causes TDP-43 cytoplasmic redistribution and aggregation [12, 22].

Also, the aging cellular environment may make the myofiber susceptible to a newly invading virus, or may allow cytopathic manifestation of a virus, or a vertically transmitted genomic endogenous virus such as a retrovirus dormant for years, such as HTLV1, may start to be transcribed due to the age-modified milieu [16]. Endogenous retroviruses (ERVs, genomic remnants of ancient viral infections, most inactive and non-infectious) are mutually reinforcing with TDP-43 proteinopathies regarding neurodegeneration [17, 26]. Moreover, aging may favor both ERVs expression and TDP-43 proteinopathy [26].

However, no definite evidence for a viral etiology of IBM has been established [27]. Mumps virus was described as a potential IBM cause, later questioned in immunohistochemical studies [28]. IBM patients have an increased prevalence of hepatitis C virus (HCV) or human lymphotropic T virus-1 (HTLV1) [69–71]. The relationship between HCV and TDP-43 is yet to be clarified. TDP-43 binds YB (Y-box-binding protein-1), a host factor involved in HCV capsids assembling, and TDP-43 knockdown significantly decreased HCV replication [19]. The persistent HCV-related IFN upregulation and lymphocyte exhaustion may in fact contribute to the chronic myopathy in HCV [4]. TDP-43 facilitates HBV gene expression stimulating its transcription and assembly of protein complexes [12]. Furthermore, the clinical picture of IBM patients with HCV is different from the one of patients with IBM and HIV; therefore, no unique mechanism links a chronic viral infection to IBM [20].

Most of the HIV-positive patients with myositis had overlapping features of PM and IBM, which clinically progress to IBM, and most of them have anti-c1NA antibodies and rimmed vacuoles [20]. TDP-43 suppresses HIV-1 transcription by binding HIV-1 long terminal repeat [72]. Knocking down TDP-43 with siRNAs in cell cultures reactivates HIV-1 by reversing its latency [23]. Notwithstanding, HIV-1 can replicate in human immune cells independent of TDP-43 [73]. In viral-associated IBM in HIV and HTLV-1, the viral antigen is not present in myofibers but in the T cells and macrophages instead [1]. HIV infection can induce T cells immune senescence [74]. Thus, it is more conceivable that the virally induced senescent, IFN-ƴ producing cytotoxic CD8+ T cells lead to IBM.

**Fig. 1:**
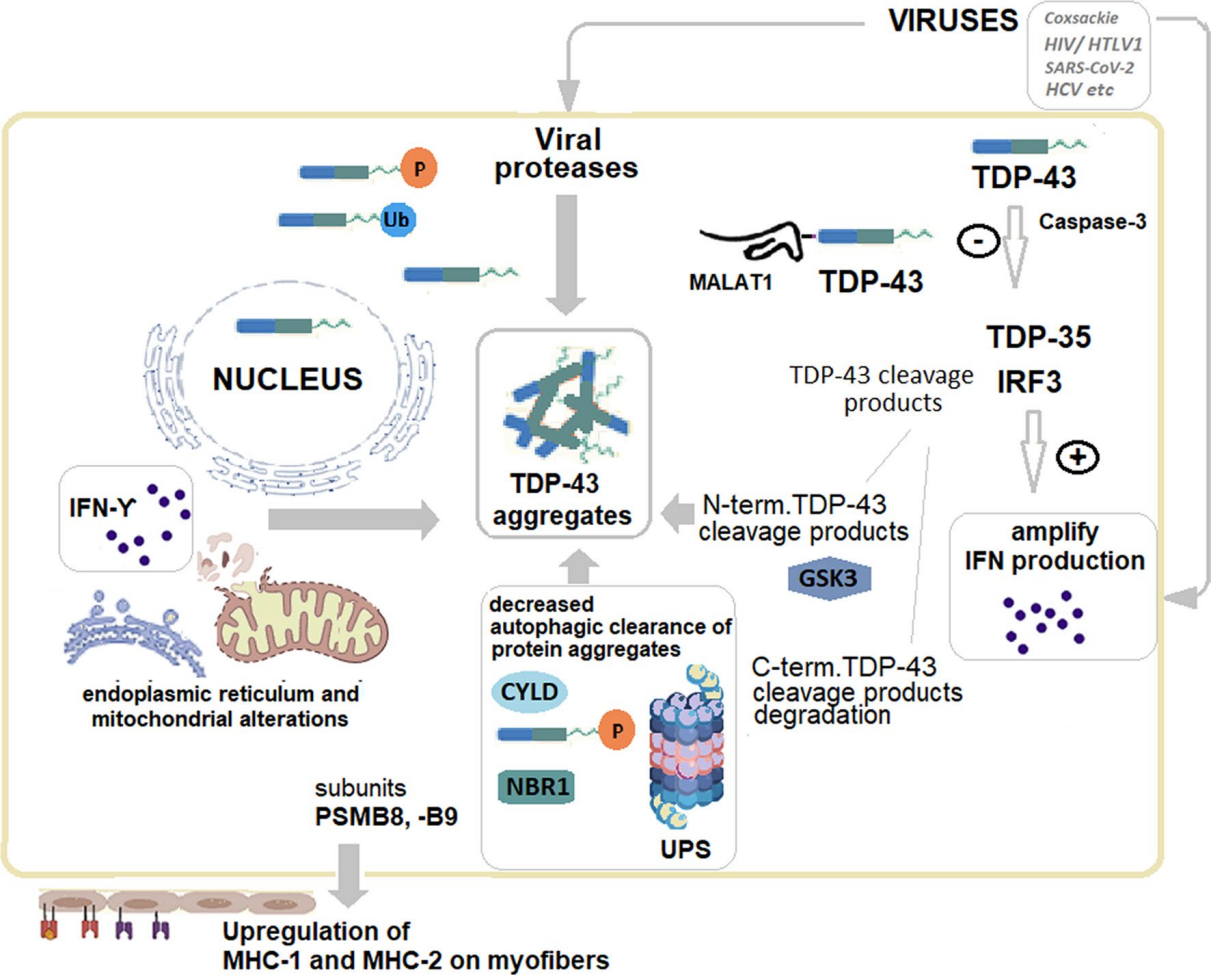
Regulation of TDP-43 in viral infections: potential implications for cellular processes in IBM pathogenesis

Legend: In IBM, TDP-43 becomes mislocalized and accumulates in the cytoplasm of cells, leading to protein aggregation and disruption of many cellular processes. Into the myofibers, in basal conditions, TDP-43 is bound by the long-non-coding RNA (lncRNA) Malat1 (metastasis-associated lung adenocarcinoma transcript-1). Malat1 binding prevents the TDP-43 cleavage, mediated by activated caspase-3, to generate TDP-35 and IRF3 (IFN regulator factor 3). TDP-35 amplifies the IFN production by degrading the negative regulator of IRF3. Generally, viral infections result in reduced expression of Malat1, promoting antiviral IFN production. However, certain viruses (Coxsackie B, hepatitis C, HIV, HTLV-1, SARS-CoV2, etc.) increase Malat1, delaying and decreasing IFN-1 production. Nevertheless, the implications for IBM pathogenesis are still hypothetical. GSK3 (glycogen synthase kinase 3) similarly enhances IFNγ and pro-inflammatory cytokines production, phosphorylating TDP-43 and promoting TDP-43 aggregation. After caspase-induced cleavage, the N-terminal cleavage product of TDP-43 may form protein aggregates, while the C-terminal cleavage product is degraded by proteasomes. Autophagy receptors may become viral targets. Amongst these autophagy receptors, NBR1 (neighbor of BRCA1), a ubiquitin-binding scaffold protein, increases in viral infections, and NBR1 accumulates and is abnormal in IBM muscle. Defects of autophagy and ubiquitin–proteasome system (UPS) result in proteostasis impairment and abnormal sarcoplasmic protein aggregation. TDP-43 is involved in the viral-induced IFN response, inducing mitochondrial and endoplasmic reticulum damage, and activating mitochondrial unfolded protein response. IFN gamma plays a major role in these processes.

IBM has been reported to be induced by Covid-19 in a 54-year female patient with diabetes mellitus and hyperlipidemia on statins [75]. Also, an axial paraspinal myopathy was reported in Covid-19 [76], and paraspinal myositis may be a feature of IBM [27]. However, long-term consequences of SARS-CoV2 infection, including muscular involvement, are starting to be recognized [77]. After COVID-19, the prevalence of myositis-specific antibodies and myositis-associated antibodies increases [78]. Possible mechanisms include type I IFN pathways, NLRP3 inflammasome activation, or a previous exposure to common coronaviruses [79]. SARS-CoV-2 impairs the stress granules (SGs) disassembly, and the SARS CoV-2 nucleocapsid N protein binds the SG-related amyloid proteins, favoring aggregation [24]. Also, SARS-CoV-2 spike S1 protein receptor binding domain (SARS-CoV2 S1 RBD) attaches to TDP-43 RRM at the viral surface, initiating aggregation [39]. TDP-43 is aggregated and hyperphosphorylated in SARS-CoV2 patients [12]. Also, the SARS-COV2 nucleocapsid N protein phosphorylation is mediated by GSK3, delaying the IFN-1 response [33] (Fig. 1).

Muscle weakness or fatigue frequently persists over 6 months after SARS-CoV2 infection, accompanied by electrophysiological myopathic changes [29, 80]. Post-acute COVID-19 sequelae (PASC) may affect 1/3 up to 2/3 of COVID-19 patients [30, 81]. PASC may be shaped by factors like endothelial damage, immunosenescence, mitochondrial alterations, and possibly by higher viral burden, and others [82]. In a longitudinal multi-omics study, SARS-CoV2 viremia, reactivation of latent viruses such as cytomegalovirus (CVM) and Epstein-Barr virus (EBV) and post-acute expansion of cytotoxic T cells were amongst the factors associated with PASC [81]. A particular PASC proinflammatory immune endotype, enriched with cytotoxic effector signatures in CD8+ and NK, has been identified [81]. It is tempting to speculate that co-infection with other viruses such as CMV could “flatten” the IFN-α initial production and stimulate persistent CD8+ T cells with IFN-ƴ production in long-Covid.

Moreover, not only cytotoxic CD8+ T cells, but also the plasma cell infiltrate from the muscles of IBM patients has a distinct B cell receptor repertoire, different from DM and PM, reflecting features of antigen-driven selection and differentiation [83]. It could be speculated that the T cells and plasma cell expansions may reflect linked recognition of common antigens, which needs further study in IBM [1, 83]. Moreover, in some IBM patients, there is an increased population of large granular T lymphocytes (T-LGL) characterized by augmented expression of surface molecules KLRG1 and CD57 [1]. The expanded T-LGL in IBM are rather secondary, “reactive,” with a senescent-like profile, associated with inhibitory NK cell receptors and increased inflammatory and cytotoxic features [84]. Of interest, HIV-1 infection is also a risk factor for the evolution of clonal T-LGL disorders [85].

In IBM as in other autoimmune diseases, immunoaging may come with an increased risk for autoimmunity, possibly the price to pay to preserve some of the immune competency [86]. The virally induced senescent, IFN-ƴ producing cytotoxic CD8+ T cells may be the ones involved in IBM, in a predisposed host.

### Genetics in IBM and viral infections

Susceptibility genes for IBM include HLA DRB1*03:01, 01:01, and 13:01 alleles, respectively [87–89]. The HLA-DRB1*03 allele, as a component of the ancestral HLA 8.1 haplotype, is a susceptibility factor for IIMs and many other autoimmune diseases [90]. An arginine in position 74 of the DRβ1 chain confers the allelic risk for IBM [89]. HLA DRB1*01 is also associated with rheumatoid arthritis and hematologic malignancies, all overrepresented in IBM and associating age-related stochastic accumulation of CD8+ CD28- T cells [1, 86]. HLA-DRB1 alleles expression also impacts durable control of viral replication, HLA DR B1*03:01 being associated with high HIV viremia [91, 92], while HLA DRB1*01 was associated with spontaneous viral clearance of hepatitis C [92].

HLA DRB1*13 is common for IBM susceptibility and for protection against infection with several viruses, including HIV, HCV, HBV [87]. In IBM, the HLA DRB1*13:01 was associated with the highest age of onset and the lower strength [88]. Nevertheless, intriguingly, HLA DRB1*13 was protective against autoimmune diseases such as systemic lupus erythematosus, psoriasis, systemic sclerosis, and others [93]. However, HLA DRB1*13 is also associated with a slow progression of HIV [94]. HLA DRB1 *13 is associated with the clearance of hepatitis B as well [95]. Surprisingly, HLA DRB1*13 is neuroprotective, along with apoE, against age-related brain changes [96]. HLA-C*14:02:01 allele was higher in IBM patients with high LGL T cell expression [84]. HLA-C*14:02 allele was also associated with a T cell response in HIV-1 infection, which was nevertheless non-protective for the viral infection [97].

HLA-F, found in IBM and Sjogren’s syndrome, also elicits antiviral responses through activation of the KIR3DS1+ NK cells [98, 99].

A bioinformatic analysis identified 10 genes in IBM, most of them involved in immune mediated and infectious diseases, including *CCR5* (encoding the human C–C motif chemokine receptor type 5), *IRF8* (interferon regulatory factor 8), HLA DRB1, *CD74*, and others [100]. CCR5 is also common for IBM susceptibility and for antiviral protection [88]. CCR5, expressed by tissue-resident memory T cells, is centrally involved in immunosurveillance, in inflammatory, autoimmune, and neoplastic disorders [101]. CCR5 also serves as an HIV co-receptor [102]. Similarly, a bioinformatic analysis found common molecular mechanisms between IBM and Sjogren’s syndrome, related to viral infection and antigen processing/presentation [99]. Amongst the 29 common genes identified, *PSMB9* encodes the immunoproteasome B9, while *CD74* encodes the cluster of differentiation 74 (also called HLA class II invariant gamma chain), a transmembrane glycoprotein contributing to antigen presentation [98]. CD74, as a key molecule of macrophage activation, involved in IFN-I and IFN-γ associated pathways in IBM and in the interaction between myofibers and macrophages in IBM [103]. CD74 interacts with the macrophage migration inhibitory factor, and CD74 upregulation contributes to immune damage during HIV infection [104].

To conclude, many genes predisposing to IBM are also involved in antiviral defense, mostly in generating interferon type I and type II.

### Therapeutic strategies involving TDP-43 in IBM

IBM currently has no effective long-term therapy [1]. Immunosuppression later during the disease course did not improve IBM, and T cell depletion did not prevent vacuole formation and disease progression [10]. Moreover, immunosuppressive therapies may sometimes reveal an underlying chronic infection [20]. Therefore, HIV testing is advisable mostly in PM/IBM overlaps [20]. Also, pan-JAK inhibitors in aged mice alleviated the senescence—associated secretory phenotype but may also reactivate latent viruses [55]. Trials of immunosuppressive therapies in IBM have been recently nicely reviewed [105]. Immunosuppression is not routinely advised unless IBM is rapidly progressive or associated with other autoimmune diseases [105].

Followed both inflammatory and myodegenerative pathways presumed to be involved in IBM pathogenesis [105]. Most studies addressed inflammation or the involvement of T cells. Alemtuzumab (against CD52), natalizumab, anti-TNF alpha such as infliximab or etanercept, or IL-1 inhibitors as anakinra and canakinumab showed modest or no improvement [105], Rapamycin (sirolimus) targets mTOR important in IL-2 immune responses and protein metabolism (NCT04789070) [105]. Novel therapeutic avenues involve anti-KLRG1 antibodies, targeting a surface marker of the highly differentiated CD8T cells (NCT04659031) [84, 105]. Moreover, in HIV, the KLRG1 expression on NK cells correlates with HIV transcription, and targeting KLRG1 on NK cells potentially aids in elimination of HIV-infected cells [106]. Therapies against myodegeneration have recently become targets in clinical trials (arimoclomol, bimagrumab, follistatin, oxandrolone, rapamycin) [105].

Possible future directions may address other pathways. The attempts to reduce TDP-43 level led to muscle weakness and defective regeneration in myopathy models [6]. However, in neurological disorders such as ALS and other TDP-43-associated diseases, affecting skeletal and cardiac muscles besides neurons, there are several TDP-43 directed therapies [107, 108]. In ALS inhibition or deletion of cGAS and STING prevents TDP-43-induced upregulation of NF-kB and IFN type I [107]. Nevertheless, the neurological and muscular effects are not completely superposable [6].

Research including new therapies and repurposing for IBM some drugs used with other indications could serve as directions for the future [109]. Future therapeutic approaches could include inhibition of TDP-43 aggregation, the TDP-43-mitochondria association, proteasomal degradation of cytoplasmic TDP-43, or reducing TDP-43 aggregation-induced cell stress [37, 38, 110, 111]. Drugs stimulating the proteasome, such as chlorpromazine and other phenothiazines, methylene blue as a structural analogue of chlorpromazine and pyrazolones may target proteotoxic disorders [112]. The efficacy of zetomipzomib (KZR-616), a selective inhibitor of the immunoproteasome, is being studied in a phase 2 controlled multicenter study for active PM and DM [113, 114]. GSK3 inhibition decreases TDP-43 aggregation [34]. Lithium inhibits GSK-3 and induces autophagy, which may be relevant for IBM [115]. Also, lithium protected synapses from HIV-1 Tat-induced neuronal loss, in cultures and may be neuroprotective in HIV [116, 117]. Some other GSK3 inhibitors (including famotidine, naproxen, olanzapine, curcumin-all sterically hindering the enzyme binding pocket) may be tested for repurposing in IBM [33]. Also, regulating CYLD could be tested as a possible a therapeutic strategy in IBM [63].

The connection between a chronic viral infection and IBM deserves to be investigated further. There are questions waiting to be answered. Which factors are involved in transforming acute viral myositis into chronic inflammatory idiopathic myopathy? And moreover, why do some aged patients develop after a viral infection an IIM, for instance an anti-synthetase syndrome, and others an IBM? For instance, in HIV infection, what conditionate the switch from a PM phenotype to an IBM one? [4]. Serial studies in patients with chronic viral infections and signs of myopathy and/or sarcopenia would probably shed light on this progression, also regarding the progression to immunosenescence, mitochondrial dysfunction and proteinopathy, and the role of TDP-43 in this setting.

## Conclusions

TDP-43 is important in preventing the dsRNA-induced IFN responses [13]. Viral infections may disrupt TDP-43 solubility and function, leading to its accumulation and lack of splicing regulation. The phenotypic differences between several IBM subtypes may be conditioned, besides genetic predisposing factors and age, also by environmental triggers such as certain viruses, and by epigenetic regulators [65]. Malat1 upregulation in certain viral infections may contribute to a protracted immune response [80].

Finding early disease markers and untangling mechanisms after a viral injury could inform whether there is a window of opportunity for the anti-inflammatory therapy, hopefully stopping or slowing the plethora of accompanying proteostasis, mitochondrial, and metabolic defects. Certain viruses, high viremia, coinfections, reactivation of latent viruses, and post-acute expansion of cytotoxic T cells may all contribute to IBM, mainly in an age-shaped immune landscape, with CD8+ T cells with IFN-ƴ production. In most such cases, the virally induced senescent, IFN-ƴ producing cytotoxic CD8+ T cells are the ones involved in IBM, in a genetically predisposed host. Immunophenotyping IBM patients to identify elevated CD8+ CD57+ populations may help stratify patients with prognostic and possibly therapeutic implications [84]. Identifying pathogenic mechanisms may lead to the identification of potential new treatments or to drug repurposing to improve the outcome in this debilitating disease.

## Data Availability

No datasets were generated or analysed during the current study.

## Abbreviations

aa: Amino acid
ALS: Amyotrophic lateral sclerosis
BRCA1: Breast cancer susceptibility gene 1
Anti-cN1A: Antibodies against the cytosolic 5′-nucleotidase 1A
CANDLE syndrome: Chronic atypical neutrophilic dermatosis with lipodystrophy and elevated temperature
cGAS: Cyclic GMP–AMP synthase
CHCHD10: Coiled-coil-helix-coiled-coil-helix domain-containing protein 10
CMV: Cytomegalovirus
CYLD: Cylindromatosis, a deubiquitinating enzyme that negatively regulates signal transduction pathways, such as NF-kB signaling pathways
DM: Dermatomyositis
dsRNA: Double-stranded RNA
EBV: Epstein–Barr virus
ERV: Endogenous retroviruses
GBP2: Guanylate-binding protein 2
GSK3: Glycogen synthase kinase 3
HCV: Hepatitis C virus
HIV: Human immunodeficiency virus
hnRNP: Heterogeneous nuclear ribonucleoprotein
HSPs: Heat shock proteins
HTLV1: Human T-cell leukemia virus type 1
IFN: Interferon
IBM: Inclusion body myositis
IIMs: Idiopathic inflammatory myopathies
iPS: Immunoproteasomes
IRF: Interferon regulatory factor
lncRNA: Long non-coding RNA
Malat1/MALAT1: Metastasis-associated lung adenocarcinoma transcript-1
MDA-5: Melanoma differentiation-associated protein 5
MHC: Major histocompatibility complex
miRNA: MicroRNA
mRNA: Messenger RNA
NBR1: Neighbor of BRCA1
NF-kB: Nuclear factor kappa B
NK: Natural killer cells
NLRP3: NOD-, LRR-, and pyrin domain-containing protein 3
PASC: Post-acute sequelae SARS-CoV-2 infection
PM: Polymyositis
PSMB8: Proteasome subunit beta type-8
Rbck1: RanBP-type and C3HC4-type zinc finger-containing protein 1
RBP: RNA-binding proteins
RIG-I: Retinoic acid-inducible gene-I
RNA: Ribonucleic acid
RRM: RNA-recognition motif
SARS-CoV-2: Severe acute respiratory syndrome coronavirus 2
SARS-CoV2 S1 RBD: SARS-CoV**-**2 spike S1 protein receptor binding domain
SAMHD1: Sterile alpha motif domain and histidine-aspartate domain-containing protein 1
SGs: Stress granules
ssRNA: Single-stranded RNA
STAT: Signal transducer and activator of transcription
STING: Stimulator of interferon genes
TARDBP: TAR-DNA-binding-protein 43 (transactive response DNA-binding protein of 43 kDa)
TDP-43: TAR-DNA-binding protein 43
TEMRA: Effector memory T cells re-expressing CD45RA
TRIM21: Tripartite motif containing 21
UPS: Ubiquitin-proteasome system
YB: Y-box-binding protein-1
